# A simple protocol for the production of highly deuterated proteins for biophysical studies

**DOI:** 10.1016/j.jbc.2022.102253

**Published:** 2022-07-12

**Authors:** Jess Li, R. Andrew Byrd

**Affiliations:** Center for Structural Biology, Center for Cancer Research, National Cancer Institute, Frederick, Maryland, USA

**Keywords:** deuteration, protein expression, NMR, SANS, isotopic labeling, DEER, EPR, DEER, double echo electron resonance, SANS, small-angle neutron scattering

## Abstract

Highly deuterated protein samples expand the biophysics and biological tool kit by providing, among other qualities, contrast matching in neutron diffraction experiments and reduction of dipolar spin interactions from normally protonated proteins in magnetic resonance studies, impacting both electron paramagnetic resonance and NMR spectroscopy. In NMR applications, deuteration is often combined with other isotopic labeling patterns to expand the range of conventional NMR spectroscopy research in both solution and solid-state conditions. However, preparation of deuterated proteins is challenging. We present here a simple, effective, and user-friendly protocol to produce highly deuterated proteins in *Escherichia coli* cells. The protocol utilizes the common shaker flask growth method and the well-known pET system (which provides expression control *via* the T7 promotor) for large-scale recombinant protein expression. One liter expression typically yields 5 to 50 mg of highly deuterated protein. Our data demonstrate that the optimized procedure produces a comparable quantity of protein in deuterium (^2^H_2_O) oxide M9 medium compared with that in ^1^H_2_O M9 medium. The protocol will enable a broader utilization of deuterated proteins in a number of biophysical techniques.

Perdeuteration coupled with isotope labeling has been widely applied and greatly appreciated in the past 2 decades for protein NMR spectroscopy (1, 2, 3, 4, 5). Conventional NMR spectroscopy has benefited from uniformly labeled ^15^N, ^13^C protein samples, and, when these labeling patterns are combined with replacement of all nonexchangeable protons with deuterons to create a perdeuterated background, it is possible to study very large proteins and protein complexes (6, 7, 8, 9, 10). Furthermore, extension of the labeling pattern to include specific methyl group labeling (^13^CH_3_), within the context of an otherwise perdeuterated background, enables a still wider range of studies into the structures, binding interactions, and conformational dynamics of proteins and protein complexes (11, 12, 13). The combined effect of these sophisticated isotope labelings has permitted researchers tremendous breadth in gaining insights of biological systems and their dynamic interactions, using NMR spectroscopy as the main tool. In recent years, as the biophysical research toolbox expands rapidly, both in terms of technology and instrumentation, it has become very clear that deuteration of proteins and other biomolecular samples will greatly aid in high-quality outcomes for other biophysical applications, particularly small-angle neutron scattering (SANS) (14, 15, 16, 17), pulsed double echo electron resonance (DEER) techniques (18, 19, 20), and neutron reflectometry (21, 22). The general requirement is to effectively remove, or make nonresponsive, a component of the measurement as a result of perdeuteration, either through contrast matching as in SANS (23) or elongation of relaxation times *via* removal of dipolar coupling as in DEER and NMR (3, 7, 24, 25). The SANS and NMR methods are further enhanced in the context of segmental labeling (26, 27, 28, 29), wherein portions of a macromolecule may be prepared with deuteration for SANS (and/or isotopic labeling for NMR) and the other portions are protonated. Hence, the need for an efficient, reproducible protocol for high-level deuteration is broadly applicable to numerous experimental methods.

Although highly desired, deuterated (^2^H) sample preparation has been notoriously problematic. Most researchers find it arduous and inconsistent. Bacterial cells (*Escherichia coli*) are the preferred host, as insect and mammalian cells are difficult to culture in a D_2_O medium (30, 31, 32). Even bacteria can be difficult to adapt to grow in high levels of D_2_O (33). Conventional procedures are time consuming and labor intensive, and the yield of deuterated samples, where the percentage labeling is >90%, can be very low. In our laboratory, we previously examined two commonly used methods: (1) medium switch prior to induction and (2) multistep media switch adaptation. In method (1), cells were grown in LB medium to the appropriate density for induction (*A*_600_ ∼ 0.8), pelleted by centrifugation, resuspended in 100% D_2_O medium, and induced immediately. This method often leads to complete cell death during induction, due to lack of proper adaptation. If cells do survive, the level of protein expression is very low. In method (2), the multistep media switch adaptation, cells were grown and transferred, stepwise, from LB to 50%, 75%, and 100% D_2_O media, which discards considerable isotopic media in the adaptation phase. This method also results in low protein expression due to multiple centrifugation and resuspension steps, rendering a generally unhealthy cell culture. The approximate yield of deuterated proteins from these methods is less than 25% of the yield from our current protocol reported here. When expression efficiency is low, the combined cost of D_2_O, ^15^N/^13^C sources, and/or specific ^13^C-labeled metabolic precursor compounds can become very high, thus limiting widespread application and realization of the full potential.

We report a highly reproducible, user friendly, time and isotope efficient, and high percentage labeling deuteration procedure that can support the ever-expanding applications in the extended communities. Our protocol eliminates many of the common roadblocks: (1) the carryover of any rich medium (LB, Superbroth, etc.) into the final culture; (2) multiple media transfers during cell training, which involves repetitive cell pelleting, resulting in long recovery time for bacterial cells and unhealthy growth; (3) extended growth period that requires lengthy in-person monitoring; (4) an unhealthy cell growth curve that often leads to low expression and even cell death.

## Results

**Figure 1:**
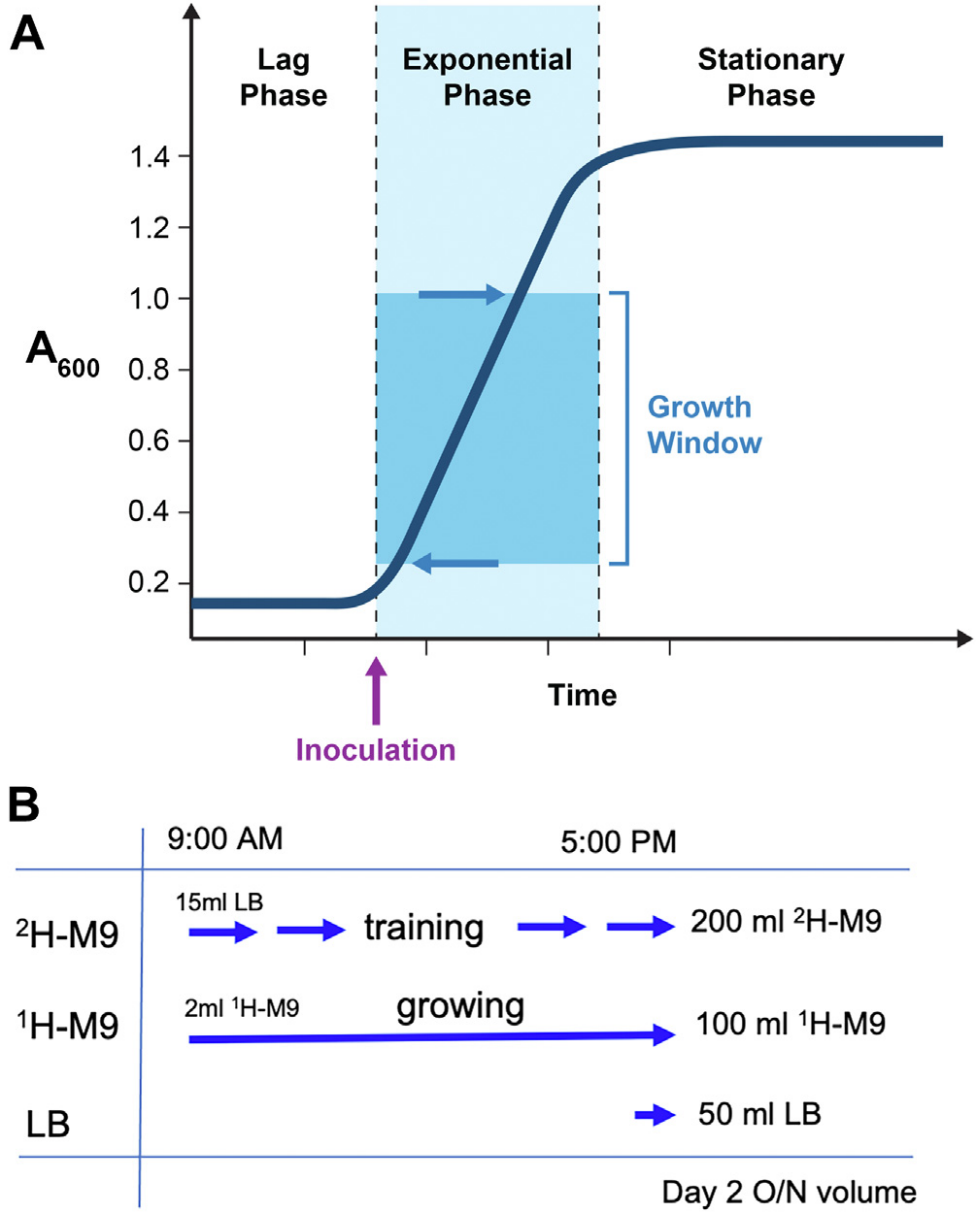
**Nominal growth curve and comparative day 2 growths on different media.***A*, nominal bacterial growth curve. Growth is monitored by the A_600_, rather than the typical doubling time. *A*_600_ from 0.2 to 1.0 is the preferred growth window. Cell density at initial inoculation and each expanding step is maintained at ~0.2 to 0.3 for healthy growth, time-saving, and fast amplification. *B*, a general timeline for day 2 cell culture in all three media. ^2^H-M9 culture goes through training as described in Figure 2. For ^1^H-M9, a small 2 ml starter culture is grown during the day to reach *A*_600_ ~0.2 to 0.3, then added into 100 ml media for overnight growth. For LB growth, inoculate a dozen colonies directly into 50 ml media for overnight. Note, day 2 overnight culture volumes are different for each media reflecting the cell density in each media after overnight growth, with LB at the highest, ^2^H-M9 at the lowest. By adjusting the volume accordingly, the initial cell density on day 3 is ensured to be around 0.2, thus maintaining the exponential growth window.

The key to successful cell growth in deuterated medium (^2^H-M9; for abbreviations and media descriptions see Experimental procedures) is maintaining cell density in the exponential phase (Fig. 1*A*). The protocol keeps *A*_600_ at or slightly above 0.2 each time a culture is initiated or transferred or following a dilution. This approach ensures that the cells are the most healthy and viable to adapt into a higher D_2_O percentage environment. The protocol is described for production of 1 liter ^2^H-M9 expression medium. LB and ^1^H-M9 cultures do not require training (adaptation). A modified simple protocol for expression in LB and ^1^H-M9 is described in the Supporting information and illustrated in Figure 1*B*.

**Figure 2:**
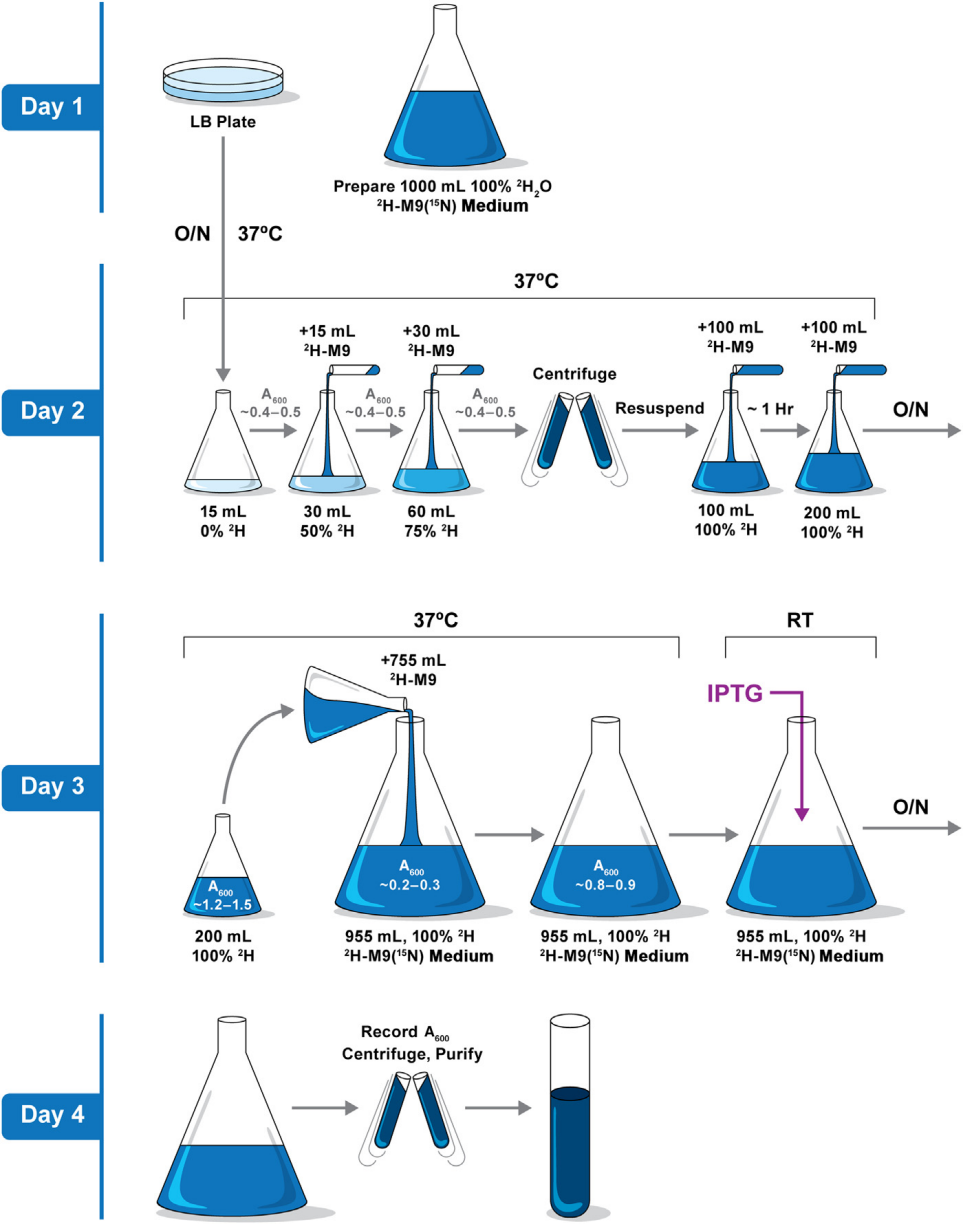
**Schematic illustration of 1** **liter**^**2**^**H-M9 expression including cell adaptation.** Variations to induction conditions are described in the text for individual protein expression.

A schematic flow chart for expression of deuterated proteins in ^2^H-M9 is illustrated in Figure 2. In general, the flask (tube) used for cell growth is 4 to 10 times the volume of the culture, which ensures proper aeration during growth. Cultures are shaken in a temperature-regulated incubator at 250 rpm. Antibiotics are chosen based on the vectors in which the genes are constructed and added to all media for a given protein expression. Carbenicillin is preferred over ampicillin for stability, and all media should be stored at 4 °C.

### Protocol

#### Day 1

1. Perform fresh transformation of plasmid DNA into BL21∗ competent cells.
2. Plate on LB agar with proper antibiotic selection.
3. Grow overnight at 37 °C.
4. Aim to produce a plate with fresh, well-isolated colonies. Frozen cell stocks are not preferred for ^2^H_2_O medium growth.
5. Prepare 1 liter of the desired labeling medium, *e.g.*, ^1^H-M9(^15^N) or ^2^H-M9(^15^N); store at 4 °C.

#### Day 2

1. Inoculate 15 ml LB media (in a 250-ml flask) with about two dozen freshly transformed colonies. The general guideline is to use enough colonies so that *A*_600_ can reach ∼0.4 to 0.5 after about 2-h growth at 37 °C.
2. Allow the initial 15 ml LB to grow to *A*_600_ ∼0.4 to 0.5, add 15 ml of ^2^H-M9 medium in the same flask to a total volume of 30 ml, which is now ∼50% in ^2^H_2_O. Continue growth at 37 °C.
3. When the 30 ml culture (∼50% in ^2^H_2_O) reaches *A*_600_ ∼0.4 to 0.5 (usually in approximately 1 h), add 30 ml of ^2^H-M9 medium to a total volume of 60 ml (in the same 250-ml flask, now at 75% ^2^H_2_O). Continue growth at 37 °C until *A*_600_ ∼0.4 to 0.5 (in approximately 1 h).
4. Centrifuge the cells (10 min, 3000*g*), discard the medium, and resuspend the cell pellet in 100 ml fresh ^2^H-M9 medium (in a 500-ml flask, now at 100% ^2^H_2_O).
5. Continue to grow at 37 °C for approximately 1 h to allow the cells to acclimate to the 100% ^2^H_2_O environment. Finally, add 100 ml ^2^H M9 medium, reaching a total culture of 200 ml. Let growth continue at 37 °C overnight.

#### Day 3

1. Record the *A*_600_ following overnight growth, which should be approximately 1.3 to 1.5.
2. Combine the 200 ml culture with the remaining 755 ml of ^2^H-M9 medium in a 4-l flask, and record the *A*_600_, which should be ∼0.2 to 0.3.
3. Continue to grow at 37 °C and monitor *A*_600_. Cells usually double in density every 2 h in ^2^H-M9; allow cells to double twice to about *A*_600_ = 0.8 to 0.9.
4. If needed, supply additives specific to the desired sample, such as precursors for custom isotope labels or prosthetic groups for posttranslational modifications or other supplementary elements.
  • For methyl labeling, appropriate metabolic precursors (*e.g.*, α-keto acids (5, 12, 34)) should be added according to manufacturer instructions. For example, to label stereospecific ^13^C-Ile, -Leu, and -Val, the appropriate kit (TLAM-I^σ1^-LV^proS^, NMR-Bio) was added to the medium 30 min prior to IPTG induction. For myr-Arf1, addition of 50 mg sodium myristate and 50 mg coenzyme A are needed for posttranslational myristoylation. Other supplementary elements, such as Zn^2+^, which is required for high expression of ASAP1 PZA, can also be added at this growth stage, 30 min prior to IPTG induction.
5. Induce protein expression, wherein the temperature and duration time will vary based on the nature of proteins to be overexpressed.
  • Generally, cultures are cooled down (while shaking) for approximately 30 min in a shaker adjusted to room temperature. Cell density will expand moderately to about 0.9 to 1.0 *A*_600_ during cool down. Induction is initiated by adding IPTG to 0.2 mM, and growth is continued at room temperature overnight. These cells are harvested in the morning of day 4. Specifically in this comparative study, the general procedure is followed for ASAP1 PH, ASAP1 PZA, and myr-Arf1; however, for Ube2g2, gp78c, and MSPDH5, cultures are maintained at 37 °C for 30 min after supplying additives and then induced with 1 mM IPTG for 3 h. Cells are harvested at the end of day 3. This 37 °C induction modification is necessary based on our knowledge and extensive testing on these proteins. We find that a significant number of mammalian proteins overexpressed in *E. coli* cells exhibit some level of toxicity, which can lead to reduced or completely abolished expression when induced at room temperature overnight. We recommend researchers test both induction conditions for their proteins of interest.

#### Day 4

1. Record *A*_600_ of the final culture and collect a 20-μl sample for SDS-PAGE analysis.
2. Harvest cells by centrifugation (10 min, 3000*g*) and either proceed with protein-specific purification or store the cell pellet at −80 °C.

As demonstration of the generality of this protocol, we provide data for the expression of six different mammalian proteins. The proteins span a wide range of protein types and sizes, comprising a moderate-size single-domain ubiquitin-conjugating E2 enzyme (Ube2g2) (24, 35, 36), an isolated domain of a more complex multidomain protein (PH domain of ASAP1) (21), a multidomain signaling protein (PZA of ASAP1) (37), a posttranslationally modified (*via* myristoylation) protein (myr-Arf1) (38), a complex multidomain protein with internal intrinsically disordered regions (gp78C) (35, 36, 39), and the amphipathic membrane scaffolding protein (MSPΔH5) used to build nanodisc membrane mimetic particles (40, 41). Each of these systems have been expressed with high levels of deuteration (≥96%), combined with either ^15^N or complex ^13^CH_3_-methyl labels. These proteins vary in size, expression level, solubility, etc., proving the flexibility and efficiency of the protocol.

**Figure 3:**
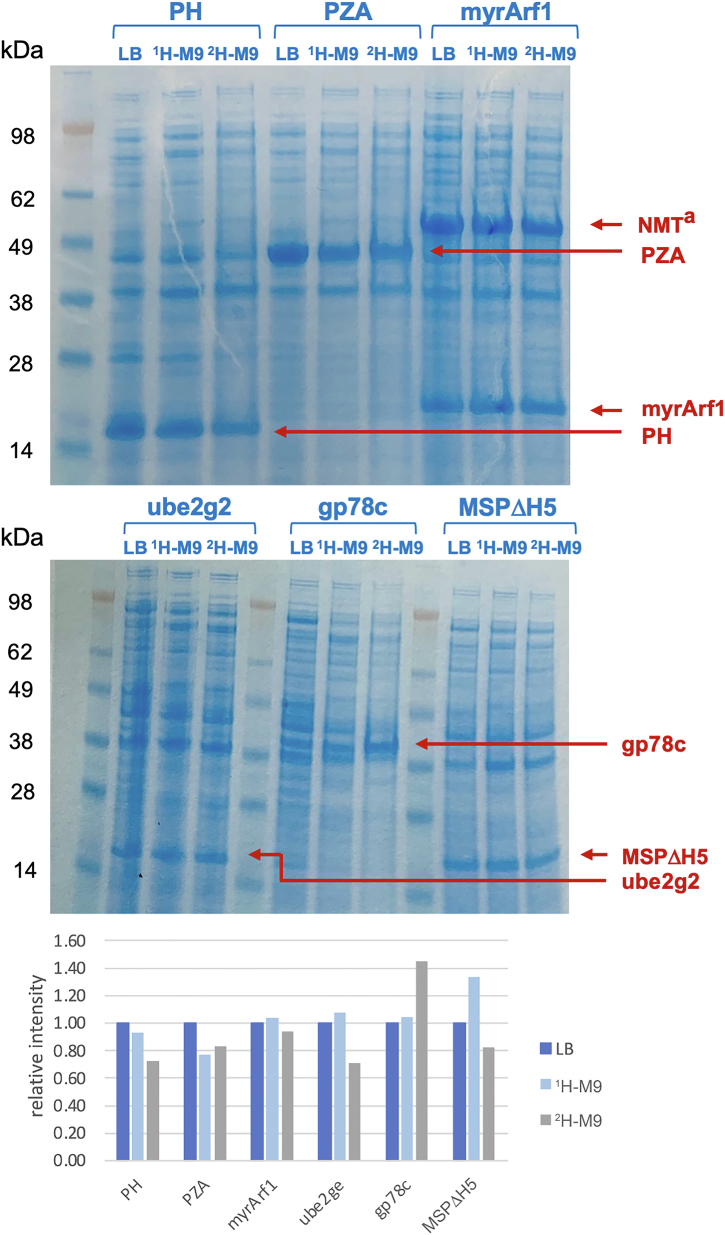
**Comparative expression of six different proteins.** Each protein is expressed in parallel using three types of media: LB, ^1^H-M9, and ^2^H-M9. IPTG-induced products are marked with *red arrows*. Samples are collected before harvest centrifugation. A 20-μl aliquot of each culture is mixed with 20 μl 2xSDS sample buffer, denatured at 95 °C for 30 min. Equal amounts of sample (20 μl) are loaded in each lane on SDS-PAGE gel. Relative intensities from digitization of bands for LB, ^1^H-M9, and ^2^H M-9 are shown for all six proteins, respectively. ^a^NMT (N-myristoyltransferase) is coexpressed with Arf1 for posttranslational modification.

The comparative performance for the six proteins is demonstrated by parallel expression in three different media: (1) LB, (2) ^1^H-M9(^15^N), (3) ^2^H-M9(^15^N) (Fig. 3). It is observed that expression levels, when following this protocol, are nearly equivalent for ^1^H-M9(^15^N) and ^2^H-M9(^15^N), and expression is only moderately reduced from LB. Each protein was purified according to the published procedures, and mass spectral data were acquired to enable determination of labeling efficiency (Table 1). In each case, the ^15^N-labeling efficiency corresponds closely to the 99% ^15^N enrichment of the nitrogen source, ^15^NH_4_Cl. Furthermore, the labeling efficiency of ^2^H, in the nonexchangeable positions, exceeds 96% for all six proteins. Each of these proteins can be prepared and purified in 5- to 50-mg/l quantities (data not shown), thus supporting a wide variety of structural and biophysical studies. In addition, we have observed that the expression level and the percentage of deuteration are not affected by other supplementary additives (such as a-keto acid precursors) in the medium, provided the carbon source (^12^C_6_^2^H_7_-glucose or ^13^C_6_^2^H_7_-glucose) is ≥97% deuterated (data not shown).

**Table 1:** Mass spectrometry data and isotopic labeling efficiencies

^*a*^ Mass represents the replacement of nonexchangeable protons with ^2^H. All exchangeable -NH, -NH_2_, -SH, -OH, and -COOH protons are calculated as ^1^H, representing back exchange during purification and mass spectrometry analyses. The mass spectrometry analyses were performed on an electrospray LC-MS that ensures full protonation of all exchangeable sites. The calculations were performed with the software package Protein Sequence Analysis, developed by Ira Palmer, NIH.

^*b*^ Predicted mass computed using the amino acid sequence and the software package Protein Sequence Analysis, developed by Ira Palmer, NIH.

## Discussion

The described procedure (Fig. 2) has been employed in our laboratory for the past decade. We confidently report that we can express the 30+ proteins from a wide range of difficult systems in highly deuterated form at similar or slightly reduced level compared with the expression in rich medium such as LB or protonated ^1^H-M9 (^15^N) medium. One of the keys to this protocol is proper attention to cell viability and avoidance of cellular and metabolic stress. Our approach deviates from conventional adaptation methods that encompass multiple steps of cell pelleting, media transfer, and resuspension. All these treatments incur additional stress and slow down, or endanger, cell growth. Unlike other published methods (42), this protocol is significantly simplified, wherein only two types of culture media (15 ml LB and 1 liter ^2^H-M9) are needed for each 1 liter production. We achieve such simplicity by starting a small, healthy LB culture and expanding (by simple dilution) the LB culture with ^2^H-M9 medium in a stepwise fashion, while maintaining the growth within the exponential phase through all media adjustments. Another advantage of the protocol is that there is no carryover of any initial LB/H_2_O media into the final expression culture, since we complete adaptation to 100% D2O at the end of day 2, which leads to the high percentage of deuteration (Table 1). In addition, the protocol is time efficient, as the total time from initial cell culture in day 2 to the harvest on the morning of day 4 is approximately 48 h. The effective use of time is achieved by two elements: (1) starting with a fresh, healthy culture in LB (H_2_O) medium and (2) adapting cells to a higher D_2_O medium by stepwise addition of ^2^H-M9 medium, thus eliminating multiple cell-pelleting steps.

Another protein deuteration method (43, 44) has encouraged high cell density growth combined with reduced culture volume. While this approach may be desirable for reducing the cost of isotope labels and D_2_O, the high cell density growth (*A*_600_ = 6.0–10.0) may incur unforeseeable cellular and metabolic stress and may not be commonly applicable for a wide range of proteins. Our protocol emphasizes healthy cell growth and is mindful of minimizing the waste of D_2_O. Overall, only 45 ml of ^2^H-M9 medium is discarded during cell adaptation per liter of growth production.

Interestingly, by observing the induced protein band intensity (Fig. 3), we find that, on average, most proteins express at a similar level across the three media. The minor changes presumably result from the characteristics of each protein. Contrary to conventional wisdom, not all proteins show reduced expression in M9 medium. Although some do show reduced expression, such as ASAP1-PH, ASAP-PZA, and myr-Arf1, others such as Ube2g2, gp78c, and MSPΔH5 express slightly better in M9 medium than in LB. One possible explanation is cell toxicity caused by low-level, basal expression in rich media (often called “leaky vectors”), while in M9 medium, basal expression is largely suppressed.

These flexible, combined labeling strategies further expand both NMR (21, 26) and SANS applications (16, 45). For NMR applications, in addition to performing ^15^N-labeling in the presence of deuteration, we can perform the full variety of methyl-specific labeling (12, 34, 46) that has become popular in NMR studies. These labeling schemes include stereospecific, uniform side-chain labeling or suppression of specific residue types. For example, the ^1^H-^13^C methyl transverse relaxation optimized spectroscopy (Fig. S1*A*) and the ^1^H-^15^N transverse relaxation optimized spectroscopy (Fig. S1*B*) spectra of ^2^H, ^15^N, ILV^proS^ ^13^CH_3_-labeled ASAP1 PZA illustrate the high spectral quality, sensitivity, and resolution that are afforded by the labeling scheme when precursors for methyl labeling are included. ASAP1 PZA is a 44.5-kDa multidomain protein that binds to membrane-associated Arf1 (21, 37, 38), and NMR studies of these interactions at a nanodisc membrane surface require high levels of deuteration, as a protonated sample would result in highly overlapping, very broad, and weak signals. The protocol and labeling scheme have also been combined with segmental labeling (26), wherein only one domain of the multidomain PZA is deuterated or ^15^N and ^13^C labeled. We have utilized highly deuterated proteins to minimize background relaxation and enable more accurate measurement of long-range, intermolecular paramagnetic relaxation effects (Zhang *et al.*, in preparation and (21)), facilitate solid-state ^1^H-detected NMR (2, 47), and DEER experiments utilizing nanodisc particles (Fig. S2, and (19)). For SANS applications, it can be advantageous to adjust the percentage of deuteration to provide optimal contrast matching in multicomponent systems (23), including protein:protein complexes (16) and membrane systems (14, 15). Since our protocol provides for complete control of ^2^H-labeling for nonexchangeable hydrogen atoms, it will be possible to fine-tune the level of deuteration. Adjustment of the final ^2^H_2_O concentration and the ratio of ^1^H_7_-glucose to ^2^H_7_-glucose in the medium can achieve a specific level of deuteration, which can be readily monitored by mass spectrometry of the expressed protein.

The protocol has proven general and enables the use of deuteration to enhance NMR and other biophysical methodologies. The protocol should facilitate and expand the application of perdeuteration in a range of biophysical experiments and enable a broad range of systems to be examined in exquisite detail. Such studies will lead to better functional understanding and mechanistic detail in many biological systems.

## Experimental procedures

### Protein expression

Each of the six proteins were expressed in *E. coli* BL21 star (DE3) cells according to the protocol presented in Figure 2. Any adjustment of conditions followed published procedures for the individual protein, as noted in Results.

### Mass spectrometry

Mass spectrometry data were acquired on an Agilent 6130 Quadrupole LC/MS System (Agilent Technologies, Inc) equipped with an electrospray source, operated in positive-ion mode. Separation was performed on a 300SB-C3 Poroshell column (2.1 mm × 75 mm; particle size 5 μm). The analytes were eluted at a flow rate of 1 ml/min with a 5 to 100% organic gradient over 5 min and holding the organic phase A for 1 min. Mobile phase A contained 5% acetic acid in water and mobile phase B was acetonitrile. Data acquisition, data analysis, and deconvolution of mass spectra were performed using Open Lab Chem Station Edition software (version C.01.05). Samples of purified proteins were typically 5 μl of a 5 μM solution.

### Materials

Isotopically labeled compounds were obtained commercially from the following sources:

Cambridge Isotope Laboratories: deuterium oxide (^2^H_2_O, Product no. DLM-4), a-keto acids (product no. CDLM-7317, CDLM-7318, etc.).

Millipore Sigma/Isotech: ^15^N-ammonium chloride (product no. 299251), ^13^C_6_^2^H_7_-glucose (product no. 552151), ^12^C_6_^2^H_7_-glucose (product no. 552003).

NMR-Bio: TLAM-I^δ1^LVpro^S^, TLAM- I^δ1^MT, and other specialty labeling kits; for residue type and stereospecific ^13^CH_3_-labeling, see http://www.nmr-bio.com.

### Solutions and abbreviations

1. M9: minimal medium described by Neidhart *et al.* (48), where the nitrogen source is derived from NH_4_Cl and the carbon source is derived from glucose. Both the nitrogen and carbon source can be supplied with ^15^N or ^13^C. In addition, the carbon source can be supplied as ^12^C_6_^1^H_7_-glucose, ^13^C_6_^1^H_7_-glucose, ^12^C_6_^2^H_7_-glucose, or ^13^C_6_^2^H_7_-glucose (the full recipe is provided in the Supporting information).
2. ^1^H-M9: M9 minimal medium wherein the solvent is ^1^H_2_O.
3. ^2^H-M9: M9 minimal medium wherein the solvent is ^2^H_2_O (99.9% ^2^H), the nitrogen source is natural abundance ^14^NH_4_Cl, and the carbon source is natural abundance ^12^C_6_^2^H_7_ glucose (97% ^2^H).
4. ^1^H-M9(^15^N): M9 minimal medium wherein the solvent is ^1^H_2_O, the nitrogen source is ^15^NH_4_Cl (>98% ^15^N), and the carbon source is natural abundance ^12^C_6_^1^H_7_-glucose.
5. ^2^H-M9(^15^N): M9 minimal medium wherein the solvent is ^2^H_2_O (99.9% ^2^H), the nitrogen source is ^15^NH_4_Cl (>98% ^15^N), and the carbon source is natural abundance ^12^C and ^2^H-labeled ^12^C_6_^2^H_7_-glucose (97% ^2^H).
6. ^2^H-M9(^15^N, ^13^C): M9 minimal medium wherein the solvent is ^2^H_2_O (99.9% ^2^H), the nitrogen source is ^15^NH_4_Cl (>98% ^15^N), and the carbon source is ^13^C_6_^2^H_7_-glucose (99% ^13^C and 97% ^2^H).
7. ^2^H-M9(^15^N, ^13^C-methyl): M9 minimal medium wherein the solvent is ^2^H_2_O (≥99% ^2^H), the nitrogen source is ^15^NH_4_Cl (>98% ^15^N), the general carbon source is ^12^C_6_^2^H_7_-glucose (97% ^2^H), and specific metabolic precursors to label the methyl groups of Met, Ala, Val, Leu, Ile, and/or Thr are provided (34).
8. LB: *Luria Broth* medium made according to manufacturer’s prescription in ^1^H_2_O solvent. MP Biomedicals, cat. No. 3002136, formulated as 4 capsules/l in double distilled H_2_O and autoclaved.
9. LB/carb or LB/Kan: *Luria Broth* medium made according to manufacturer’s prescription in ^1^H_2_O solvent containing 100 mg/l of carbenicillin or 50 mg/l of kanamycin.
10. LB plate: Agar plate made with LB/carb or LB/kan medium.
11. IPTG: isopropyl β-D-1-thiogalactopyranoside.

## Data availability

All data are contained within the article. Copies of the datasets used and/or analyzed during the current study are available from the corresponding author on reasonable request. The software, Protein Sequence Analysis, is available from Ira Palmer, NIAMS, NIH (palmeri@mail.nih.gov), and numerous other online variants exist for computing mass from primary sequence.

## Supporting information

This article contains supporting information (48, 49, 50).

## Conflict of interest

The authors declare that they have no conflicts of interest with the contents of this article.
