# Supplementary material for: A simple protocol for the production of highly deuterated proteins for biophysical studies
Source: J Biol Chem. 2022 Jul 12;298(8):102253. doi: 10.1016/j.jbc.2022.102253 (PMC9386462; doi:10.1016/j.jbc.2022.102253)
Supplement: Supplemental Figures S1 and S2 [file mmc1.pdf]

Supporting Information

High-Level Deuteration of Proteins for Biophysical Studies

Jess Li and R. Andrew Byrd

Center for Structural Biology
Center for Cancer Research, National Cancer Institute
Frederick, MD 21702-1201

Address correspondence to: lije@mail.nih.gov and byrdra@mail.nih.gov

1. M9 media ingredients (1)

**Ingredients**	**per liter (H_2O or D_2O)**
KH_2PO_4	3.0 gm
Na_2HPO_4	6.0 gm
NH_4Cl[a]	1.00 gm
NaCl	0.53 gm
Glucose[b]	2.0 gm
Vitamin B (500x)[c]	2 ml
Divalent cations (100x)[d]	10 ml
Trace elements (100,000x)[e]	10 ul

[a] ^{15}NH4Cl is used as the nitrogen source for ^{15}N labeling
[b] For perdeuteration, replace with d_7-D-glucose; for perdeuteration and uniform ^{13}C-labeling, replace with $^{13}C_6/d_7$-D-glucose.
[C] vitamin B per 500 ml (500x stock): thiamine 3,750 mg, niacin amide 2,500 mg, biotin 250 mg, choline chloride 250 mg, pantothenic acid 250 mg, pyridoxine 250 mg, folic acid 250 mg.
[d] Divalent Cations per 1 liter (100x stock): $CaCl_2 \cdot 2H_2O$ 1.32 g, $MgSO_4 \cdot 7H_2O$ 24.6 g, $FeCl_3 \cdot 6H_2O$ 1.35 g, Citrate 4.41 g
[e] Trace elements per 1 liter (100,000x stock): $(NH_4)Mo_7O_4$ 370 mg, $CoCl_2 \cdot 6H_2O$ 714 mg, H_3BO_3, 2,470 mg, $CuSO_4 \cdot 5H_2O$ 250 mg, $MnCl_2 \cdot 4H_2O$ 1,580 mg, $ZnSO_4 \cdot 7H_2O$ 288 mg

2. LB and ^{1}H-M9 culture protocol

Procedure for Day 2 is modified for LB and ^{1}H-M9 culture as following:

<u>^{1}H-M9</u>: early morning, inoculate about one dozen fresh colonies into 2 ml ^{1}H-M9 media, grow at 37°C all day, for about 6-7 hours, till culture to be visibly cloudy, pour the entire 2 ml culture into 100 ml fresh media. Grow overnight at 37°C. In Day3, combine the overnight culture with the remaining 900 ml fresh media. Continue to grow, monitor, and induce as described for ^{2}H-M9 culture.

LB: start a 50 ml LB media in mid- or late-afternoon using about one dozen fresh colonies from a plate. There is no need for training in LB (as in ^{2}H-M9), nor an initial starter culture (as in ^{1}H-M9). Grow the 50 mL culture at 37 ºC overnight. In Day3, combine the overnight culture with the remaining 950 ml fresh media. Continue to grow, monitor, and induce as described for ^{2}H-M9 culture.

3. Mass Spectrometry Measurements

Mass spectrometry data were acquired on an Agilent 6130 Quadrupole LC/MS System, (Agilent Technologies, Inc., Santa Clara, CA) equipped with an electro-spray source, operated in positive-ion mode. Separation was performed on a 300SB-C3 Poroshell column (2.1 mm x 75 mm; particle size 5 um). The analytes were eluted at a flow rate of 1 ml/min with a 5 to 100% organic gradient over 5 minutes and holding the organic phase A for 1 minute. Mobile phase A contained 5% Acetic Acid in water and mobile phase B was Acetonitrile. Data acquisition, data analysis, and deconvolution of mass spectra ware performed using Open Lab Chem Station Edition software (version C.01.05). All of the purified proteins, in these tests and for structural studies are routinely monitored with electrospray LCMS.

4. NMR

NMR spectra were collected at 25ºC on Bruker Avance III 700, 800 or 850 MHz spectrometers equipped with TCI triple-resonance cryoprobes. All spectra were processed using TopSpin 3.6 (Bruker Biospin, Billerica, MA) and NMRPipe (2), analyzed and illustrated by NMRDraw (2) and NMRFAM-Sparky (3).

Figure S1

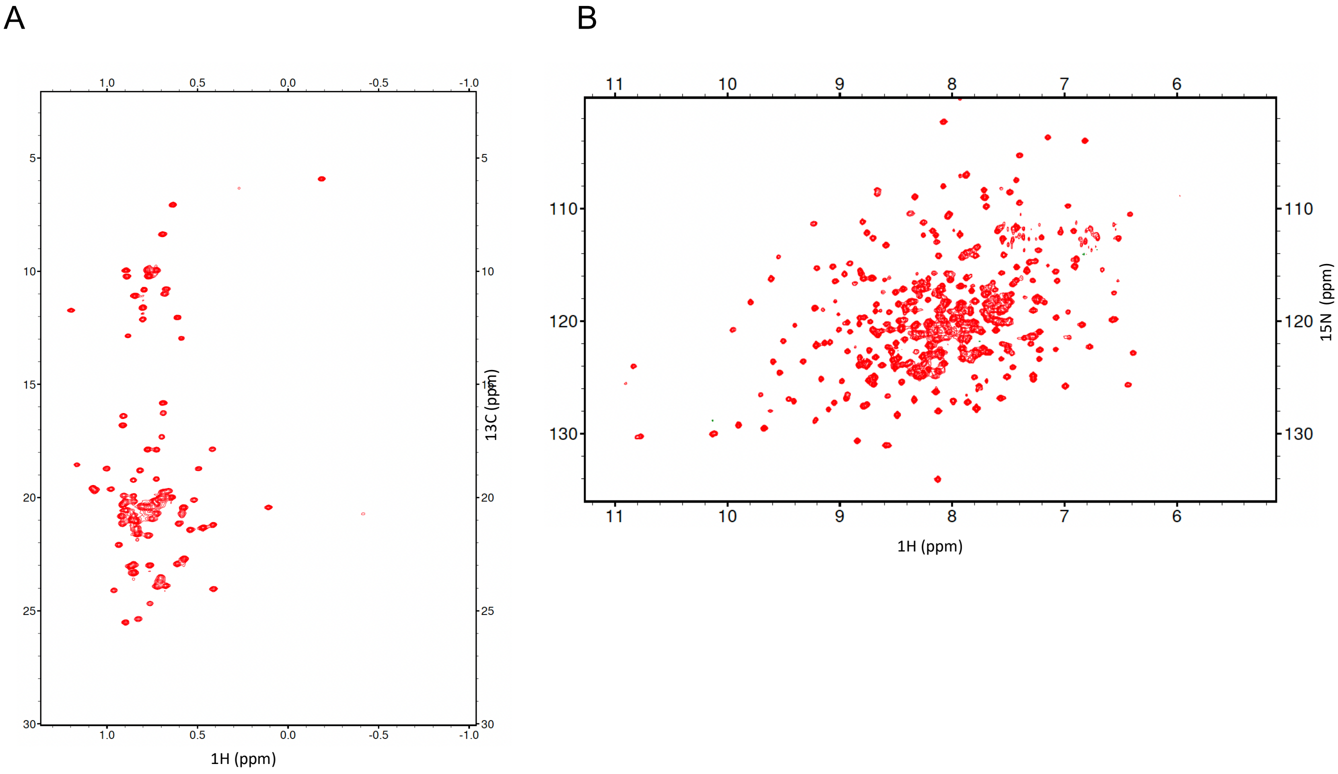

Figure S1. NMR spectra of ASAP1-PZA. A) ^{1}H-^{13}C methyl TROSY spectrum of ^{2}H, ILV^{proS} $^{13}CH_3$ labeled ASAP1 PZA. B) ^{1}H-^{15}N TROSY spectrum of ^{2}H, ^{15}N ASAP1 PZA. Spectra were acquired at 850 MHz ^{1}H frequency.

Figure S2

A

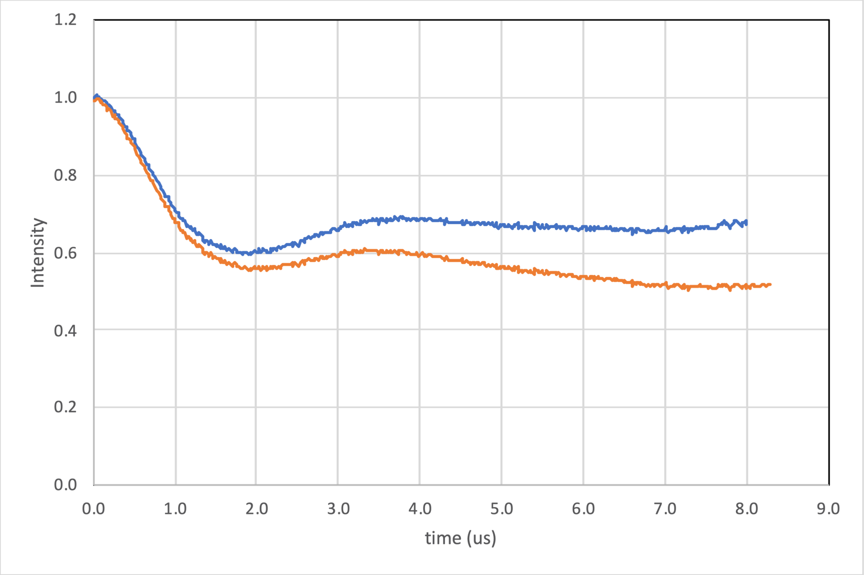

B

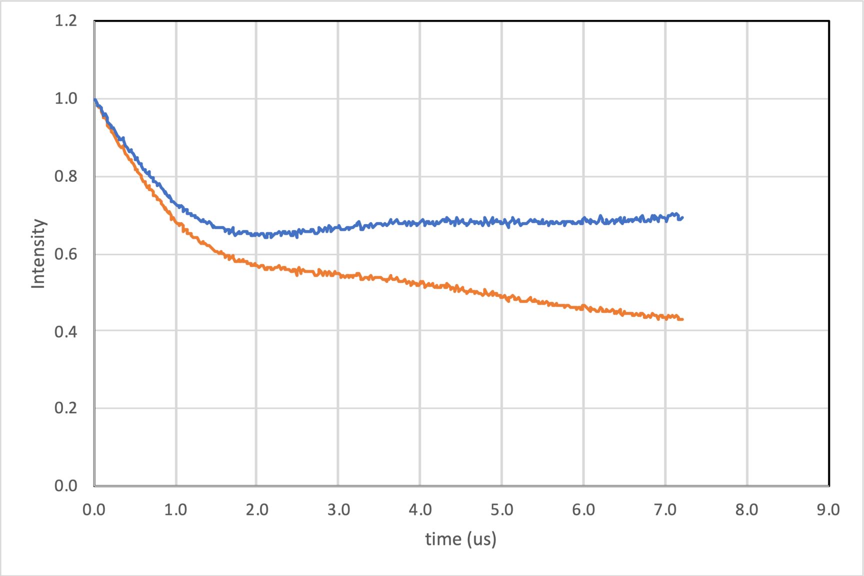

Figure S2. Time domain DEER spectra of MSPΔH5 nanodiscs formed with DMPC lipids. Raw, uncorrected data is shown in orange, and background corrected data is shown in blue. A) ND formed using deuterated DMPC-d67 lipids (Avanti Polar Lipids, Birmingham, AL, USA) and perdeuterated MSPΔH5, or B) ND formed using protonated DMPC lipids and protonated MSPΔH5. Note the significant difference in the decay rates and observed modulation of the time domain signal when both the lipid acyl chains and the MSPΔH5 belt protein are highly deuterated.

References:

1. Neidhardt, F. C., Bloch, P. L., and Smith, D. F. (1974) Culture medium for enterobacteria. *J Bacteriol* **119**, 736-747
2. Delaglio, F., Grzesiek, S., Vuister, G. W., Zhu, G., Pfeifer, J., and Bax, A. (1995) NMRPipe: a multidimensional spectral processing system based on UNIX pipes. *J Biomol NMR* **6**, 277-293
3. Lee, W., Tonelli, M., and Markley, J. L. (2015) NMRFAM-SPARKY: enhanced software for biomolecular NMR spectroscopy. *Bioinformatics* **31**, 1325-1327
